# Single-cell map of diverse immune phenotypes in the acute myeloid leukemia microenvironment

**DOI:** 10.1186/s40364-021-00265-0

**Published:** 2021-03-01

**Authors:** Rongqun Guo, Mengdie Lü, Fujiao Cao, Guanghua Wu, Fengcai Gao, Haili Pang, Yadan Li, Yinyin Zhang, Haizhou Xing, Chunyan Liang, Tianxin Lyu, Chunyan Du, Yingmei Li, Rong Guo, Xinsheng Xie, Wei Li, Delong Liu, Yongping Song, Zhongxing Jiang

**Affiliations:** 1 Department of Hematology, The First Affiliated Hospital of Zhengzhou University, Zhengzhou, Henan China; 2 Joint National Laboratory for Antibody Drug Engineering, Key Laboratory of Cellular and Molecular Immunology of Henan Province, Institute of Translational Medicine, School of Basic Medicine, Henan University, Kaifeng, Henan China; 3 The Academy of Medical Science, College of Medical, Zhengzhou University, Zhengzhou, Henan China; 4 The Affiliated Cancer Hospital of Zhengzhou University, Henan Cancer Hospital, Zhengzhou, Henan China; 5 Laboratory Animal Center, School of Medical Sciences, Zhengzhou University, Zhengzhou, Henan China

**Keywords:** Acute myeloid leukemia, Microenvironment, Single-cell RNA sequencing, Immune phenotypes, Bone marrow, Immune cells, Myeloid cells, T lymphocytes

## Abstract

**Background:**

Knowledge of immune cell phenotypes, function, and developmental trajectory in acute myeloid leukemia (AML) microenvironment is essential for understanding mechanisms of evading immune surveillance and immunotherapy response of targeting special microenvironment components.

**Methods:**

Using a single-cell RNA sequencing (scRNA-seq) dataset, we analyzed the immune cell phenotypes, function, and developmental trajectory of bone marrow (BM) samples from 16 AML patients and 4 healthy donors, but not AML blasts.

**Results:**

We observed a significant difference between normal and AML BM immune cells. Here, we defined the diversity of dendritic cells (DC) and macrophages in different AML patients. We also identified several unique immune cell types including T helper cell 17 (TH17)-like intermediate population, cytotoxic CD4^+^ T subset, T cell: erythrocyte complexes, activated regulatory T cells (Treg), and CD8^+^ memory-like subset. Emerging AML cells remodels the BM immune microenvironment powerfully, leads to immunosuppression by accumulating exhausted/dysfunctional immune effectors, expending immune-activated types, and promoting the formation of suppressive subsets.

**Conclusion:**

Our results provide a comprehensive AML BM immune cell census, which can help to select pinpoint targeted drug and predict efficacy of immunotherapy.

**Supplementary Information:**

The online version contains supplementary material available at 10.1186/s40364-021-00265-0.

## Background

Acute myeloid leukemia (AML), as a heterogeneous disease caused by several specific mutant genes (such as *FLT3*, *NPM1*, *DNMT3A*, *IDH1*, *IDH2*, *TET2*, fusion genes, and so on), is characterized by increased proliferation of abnormal myeloid progenitors with blocking terminal differentiation in BM and other tissues [1]. Rapid clonal expansion of malignant blasts harbors within the BM microenvironment, replaces heterogeneous hematopoietic cells and stromal cells, and impairs normal hematopoiesis and immune cell development [2]. The expanding malignant cells not only impairs stromal cells and hematopoiesis, but also remodels BM immune microenvironment [3]. The malignant myeloid cells can impair osteogenesis [4], HSC-progenitor transition [5], myelo-erythropoiesis [6], erythroid differentiation [7], macrophage phagocytosis [8], dendritic cell differentiation [9], T cell anti-tumor function [10], and Natural killer (NK) cell immune surveillance [11]. However, compared with other cancer types, especially solid tumor, the immune cell types, immune status, and molecular mechanisms of AML patient BM microenvironment are poorly understood.

AML microenvironment includes complex interactions between immunosuppressive cell types, cytokines, and surface stimulatory molecules. AML cells can decrease MHC-I/II expression, produce reactive oxygen species (ROS) and indolamine-2,3-dioxygenase (IDO), and increase inhibitory ligands PDL1, B7-H3 (CD276), and Galectin 9 (Gal), which lead to escape immune surveillance and T cell exhaustion [12]. Antibody-based immune checkpoint blockade for AML patients does not seem ideal [13, 14], and underlying mechanism is not very clear. NK cells, as central players of innate immune system, owns robust anti-tumor effects. Under AML stress, several mechanisms involved impairing NK cell function [11]: 1) decreased expression of IFN-γ, TNF-α, NKp30, NKp44, and NKp46, and increased inhibitory NKG2A and KIR2DL2 in NK cells [15–17]; 2) increased AML cell resistance to NK cell-mediated cytotoxicity [18–20]; 3) suppressed by immunosuppressive cell types, such as DC and Treg [21–23]. Treg is also a critical player of immune response, which can limit activation and proliferation of cytotoxic lymphocytes by secreting anti-inflammatory cytokines, competing cytokines/costimulators, and contact-dependent suppression [24]. What cannot be ignored is that roles of myeloid lineage in AML microenvironment, such as myeloid-derived suppressor cells (MDSC), macrophages, and DC. MDSC is defined as innate myeloid cells with immunosuppressive function during cancer, which can be divided into several subsets with explicit developmental trajectory [25]. MDSC-like cells also expanded in AML patients [26], but its heterogeneity and features of developmental trajectory do not benefit for screening effective specific targets, which should be identified by scRNA-seq analysis and meticulous functional assay. And malignant cells polarized macrophages towards a tumor supporting status [8, 27], which have been reported in many other tumor types [28]. In addition, DC dysfunction impairs the immune response of AML patients [29, 30]. In summary, AML blasts can systematically change the immune status of BM microenvironment to support malignant cell growth and resist immune surveillance.

In this study, we characterized the immune components of different AML patient-derived BM cells by sc-RNAseq data analysis. Different from concentrating on analysis of clonal heterogeneity and hierarchies of AML cells [31, 32], we focused on the mature immune cell types and immune status under the stress of AML and chemotherapy. To assess the composition, function, and status of immune cells, we performed scRNA-seq analysis using publicly-available datasets (GSE116256) [32]. Our analysis revealed different AML patients owned unique immune profiles, diversity of immunosuppressive DC and macrophage subsets, exhausted and dysfunctional T/NK subpopulations, and suppressive T cells with unusual developmental trajectories. Finally, we investigated several new immune cell types or status, such as TH17/Treg intermediate population, cytotoxic CD4^+^ effectors, T cell: erythrocyte complexes, activated Tregs, and CD8^+^ memory-like cells. These results have deepened our understanding of immune cell components and status, which also noted us the choice of immunotherapy strategy should be customized according to the AML patient’s specific BM immune microenvironment, rather than using trial-and-error strategies. In the foreseeable future, the most optimized and efficient immunotherapy choice will be solved by high-throughput scRNA-seq technology, based on further comprehensive investigations of tumor cell: immune cell and/or immune cell: immune cell interaction.

## Methods

### scRNA-seq datasets

The scRNA-seq datasets of AML BM cells and healthy donor BM cells were acquired from the Gene Expression Omnibus (GEO) database (GSE116256). The scRNA-seq data was acquired from BM cells of 16 AML patients and 4 healthy donors. And the information about cell preparation and single-cell transcriptome profiling can be got from the paper of *Bradley E. Bernstein* and his colleagues [32]. The GSM numbers of all these samples with other detailed information (days from diagnosis, gender, age, mutations, and so on) are listed in Supplementary Table 1.

### Quality control and data processing

Single-cell datasets of AML patients and healthy donors were integrated using “merge” function in version 3.2.2 of Seurat R package [33]. We filtered cells that have unique feature counts over 3000, less than 200, and ≥ 10% mitochondrial counts. The merged dataset was normalized using Seurat “NormalizedData” function with a global-scaling normalization method “LogNormalize”, and multiplied this by a scale factor (10,000 by default). And then scaled by performing Seurat “ScaleData” function with regression of the variation of “nCount_RNA” and “percent.mt”. Performing Seurat “JackStrawPlot” function and “ElbowPlot” function helped to select suitable dimensionality. Dimension reduction analysis was performed by Seurat “RunPCA” function, and non-linear dimensional reduction was performed by Seurat “RunUMAP” function.

### Reconstructing cell development trajectories

To explore the developmental progression of naïve CD4^+^ T cells to TH17-like cells and/or Treg cells, we used Monocle package (version 2.14.0) for reconstructing their development trajectories [34]. We extracted the dataset of naïve CD4^+^ cluster, TH17-like cluster, and Treg cluster, and then selected the cluster feature genes for the trajectory reconstruction.

### Survival analysis

The TCGA AML data (file “TCGA-LAML.htseq_fpkm.tsv”, file “TCGA-LAML.survival.tsv”, and file “gencode.v22.annotation.gene.probeMap”) were download from UCSC Xena (http://xena.ucsc.edu/) [35] and used to assess the prognostic effect of single functional genes, preference gene sets, and gene sets from cluster biomarkers. Cluster biomarkers were got through performing Seurat “FindAllMarkers” function and reporting only the positive ones. We used package “survival” and “survminer” packages to get the survival curve.

## Results

### A scRNA-seq census of AML BM immune cells pre- and post-treatment

**Fig. 1:**
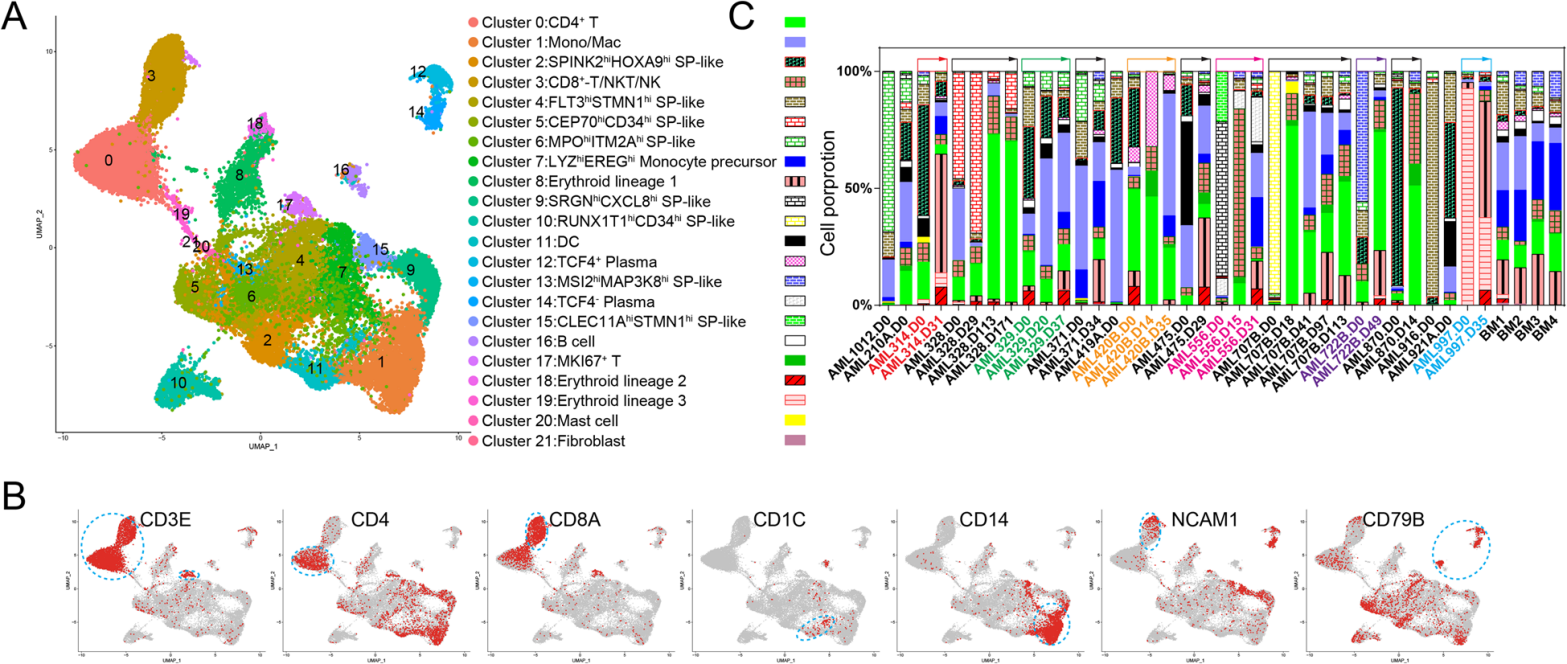
Dissection and clustering of AML patient BM cells and healthy donor BM cells. **a** The UMAP projection of BM cells from 16 AML patients and 4 healthy donors, showing the formation of 22 main clusters, including 3 for T/NK cells (Cluster 0, Cluster 3, and Cluster 17), 3 for B lineage (Cluster 12, Cluster 14, and Cluster 16), 3 for mature myeloid lineage (Cluster 1, Cluster 11, and Cluster 20), 3 for erythroid lineage (Cluster 8, Cluster 18, and Cluster 19), 9 for SP-like cells (Cluster 2, Cluster 4, Cluster 5, Cluster 6, Cluster 7, Cluster 9, Cluster 10, Cluster 13, and Cluster 15), and 1 for non-hematopoietic stromal cells (Cluster 21). Each dot represents to one single cell, and is colored according to cell cluster. **b** *CD3E*, *CD4*, *CD8A*, *CD1C*, *CD14*, *NCAM1*, and *CD79B*-expressing (expression value > 0) of across 36,477 BM-derived cells illustrated in UMAP plots. **c** Histogram of cell-type fractions for each AML patient and healthy donors’ BM cells, colored based on cell type

We hypothesized the immune phenotypes and status were remodeled by uncontrollable AML blasts, it might be identifiable in data generated from recent efforts to distinguish AML hierarchies [32]. *Bernstein* and his colleagues showed an atlas of AML cell states by scRNA-seq, and found monocyte-like AML cells suppressed T cell activity by expressing immunomodulatory genes [32]. To characterize the dynamic changes of mature hematopoietic cell lineages’ states at more refined levels, we first downloaded and explored the relevant datasets from GSE116256 (Supplementary Table 1) [32]. The scRNA-seq data from BM cells of 16 AML patients and 4 healthy donors was performed uniform manifold approximation and projection (UMAP) analysis [33]. These 36,477 BM-derived cells segregated into 22 populations (Fig. 1a). These populations were identified based on the expression of canonical marker genes for mature terminal lineages (Fig. 1b) and remarkable genes for hematopoietic stem/progenitor cells (HSPCs) or leukemia stem/progenitor cells (LSPC) (dubbed “SP-like cells”) (Supplementary Figure 1A-D).

Then we analyzed the compositions of mature hematopoietic lineages in AML samples during treatment (Fig. 1c). Compared with healthy BM samples, some AML samples (AML420B.D0, AML420B.D15, AML420B.D31; AML328.D113, AML328.D171; AML707B.D18, AML707B.D41, AML707B.D97, AML707B.D113; AML870.D14) have higher proportions of CD4^+^ T cells (Cluster 0), which indicated that chemotherapy increased the frequency of CD4^+^ T cells with wiping out malignant cells. Most untreated AML samples had a low frequency of CD8^+^-T/NKT/NK subset (Cluster 3). And the chemotherapy can increase the frequency of this cluster, especially in the second to third weeks after treatment (Supplementary Figure 1E). This trend also occurred within MKI67^+^ T cell population (Cluster 17) (Supplementary Figure 1B), LYZ^hi^EREG^hi^ monocyte precursor population (Cluster 7), and erythroid lineage 1 (Cluster 8) (Supplementary Figure 1E) in some patients. Interestingly, Mono/Mac subpopulation (Cluster 1) and erythroid lineage 2 (Cluster 18) showed two different treatment response models (Supplementary Figure 1E), which might be associated with the functional heterogeneity of mature cells, needing more detailed analysis. And the treatment could decrease the frequency of B cells (Cluster 16) initially, and increase subsequently. Of note, some AML patients had disproportional number of mast cells (Cluster 20), and treatment can decrease this population (Supplementary Figure 1E). Interestingly, analyses of AML samples from TCGA indicated that patients with high expression of signature genes of MSI2^hi^MAP3K8^hi^ SP-like population and mast cells, low LYZ^hi^EREG^hi^ monocyte precursor, DC, and MKI67^+^ T showed significantly better overall survival (Supplementary Figure 1F). And specific-gene signature of other clusters, such as CD4^+^ cluster and CD8^+^-T/NKT/NK cluster did not yield significant survival association, which is not consistent with perception of T/NK cell anti-tumor function. And these results foreboded the immune cell function might degrade or inverse in a broader sense.

AML is a high-risk hematological malignancy and show high heterogeneity with complex mutant and/or fusion gene combinations. AML cells occupies the niche of hematopoiesis, leads to ineffective hematopoiesis, induces immune dysfunction. Periphery blood (PB) samples of AML patients showed us the immune dysfunction of lymphocytes based on the analysis of flow cytometry and immune-related risk of factors, indicated that the immune signatures corrected clinical outcomes [36]. The immune status of BM microenvironment are more sensitive to reflect immune response for clearing AML blast cells compared to PB [37]. So dissecting the immune landscape of AML is important for predicting the immune status and screening suitable immunoregulatory drugs. Our analysis based on scRNA-seq of AML BM cells can detect much more details to dissect the immune landscape, and help to find new immune cell subsets and treatment strategies.

### The unique composition of DC subtypes in AML patients

**Fig. 2:**
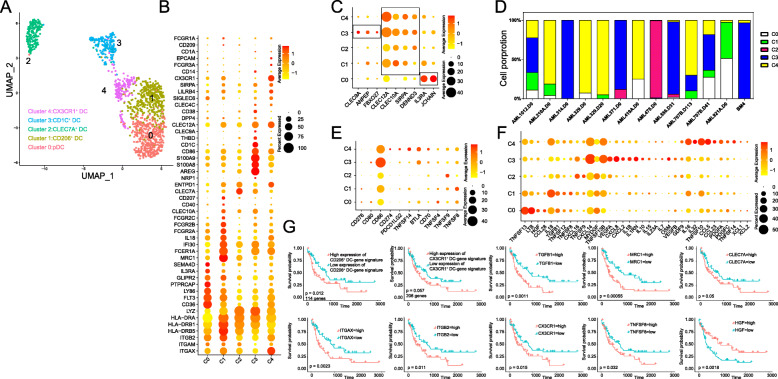
Dissection and clustering of DC-like cells in AML patients and healthy donors. **a** UMAP plot of DC-like cells from Fig. 1a-represented DC cluster. These DC-like cells can be divided into 5 subsets. **b** Expression of DC-related genes across the transcriptionally defined DC clusters. **c** Dot plot of select canonical DC-related genes (cDC1: *CLEC9A*, *ANPEP*, and *FBXO27*; cDC2: *CLEC12A*, *CLEC10A*, *SIRPA*, and *DENND3*; pDC: *IL3RA* and *JCHAIN*) differentially expressed between different DC subsets. **d** Proportion of 5 DC subsets in total DC cells in each AML patient or healthy donor. Only samples of containing ≥10 DC-like cells were represented. **e** Dot plot of differentially expressed co-inhibitory and co-stimulation molecule genes. **f** Dot plot of differentially expressed cytokine genes. **g** The Kaplan-Meier overall survival curves of TCGA AML patients grouped by specific DC subset (CD206^+^ DC and CX3CR1^+^ DC) gene sets and DC-related genes (*TGFB1*, *MRC1*, *CLEC7A*, *ITGAX*, *ITGB2*, *CX3CR1*, *CCL22*, and *TNFSF8*). + represents censored observations, and *P* value was calculated by multivariate Cox regression

To uncover the spectrum of DC heterogeneity and states, we utilized UMAP to re-cluster 1293 DC-like cells (Fig. 2a), and got 5 clusters (Table 1). Consistent with previous studies, the DC-like population derived from AML patients and healthy donors, expresses *CD11c* (*ITGAX*), *CD18* (*ITGB2*), and MHC II molecules (*HLA*-*DRB5*, *HLA*-*DRB1*, and *HLA*-*DRA*) at high levels, but *CD11b* (*ITGAM*) at a low level (Fig. 2b), which identified the DC population [38]. Cluster 0 express *CD36*, *FLT3*, *LY86*, *PTPRCAP*, *GLIPR2*, *IL3RA* (*CD123*),and *SEMA4D* at high levels, but *LYZ* at a low level, and was defined as plasmacytoid dendritic cells (pDC) [39]. Compared with other 4 clusters, CD206^+^ DC subpopulation (cluster 1) express higher levels of *MRC1* (*CD206*), *FCER1A*, *IFI30*, *IL18*, and *Fc gamma receptor II* (*FCGR2A*, *FCGR2B*, and *FCGR2C*), with an obvious *CD4*-expressing feature (Supplementary Figure 2A). Cluster 2 (dubbed “CLEC7A^+^ DCs”) is identified by high expression of *CLEC7A* and *ITGAE* (*CD103*) (Supplementary Figure 2A).

**Table 1:** Immune cell types and functions in AML BM microenvironment

Cluster	Subset	Feature genes	Anti-tumor or immunosuppressive /dysfunctional
**Mast cell**	**–**	High: *KIT*, *GATA2*, *LMO4*, *CPA3*, *TPSAB1*, *TPSB2*	Anti-tumor
**DC**	**pDC**	High: *CD36*, *FLT3*, *LY86*, *PTPRCAP*, *GLIPR2*, *IL3RA*, *SEMA4D*, *JCHAIN*, *TNFSF4*, *TNFSF13*, *LTB*, *HGF*, *CCL28*; Low: *LYZ*	Anti-tumor
	**CD206**^**+**^ **DC**	High: *MRC1*, *FCER1A*, *IFI30*, *IL18*, *FCGR2A*, *FCGR2B*, *FCGR2C*, *CD4*, *TNFSF12*, *TNFSF8*	Immunosuppressive
	**CLEC7A**^**+**^ **DC**	High: *CLEC7A*, *ITGAE*	Undefined
	**CD1C**^**+**^ **DC**	High: *AREG*, *S100A8*, *S100A9*, *CD86*, *CD1C*, *CLEC9A*, *ANPEP*, *FBXO27*	Undefined
	**CX3CR1**^**+**^ **DC**	High: *CX3CR1*, *CD8A*, *CD274*, *PDCD1LG2*, *TNFSF14*, *BTLA*, *CD70*, *TNFSF10*, *CCL5*	Immunosuppressive
**Mono/Mac**	**MS4A6A**^**high**^ **subset**	High: *MS4A6A*, *CD86*, *CSF1R*, *TGFB1*, *CCL22*	Immunosuppressive
	**CD163**^**high**^ **subset**	High: *CD163*, *CXCR4*, *IL4R*, *TGFB1*, *CCL18*	Immunosuppressive
	**MKI67**^**+**^ **subset**	High: *MKI67*, *VEGFA*	Undefined
	**MARCO**^**high**^ **subset**	High: *IL1B*, *MARCO*, *CD4*, *CD68*, *CD86*, *CD14*, *CD163*, *IL4R*, *CSF1R*, *CX3CR1*, *TGFB1*	Immunosuppressive
	**FCGR3A**^**+**^ **subset**	High: *CX3CR1*, *FCGR3A*, *CSF1R*	Anti-tumor
	**MRC1**^**+**^ **subset**	High: *MRC1*, *CSF1R*, *TGFB1*	Immunosuppressive
	**CD14**^**high**^**ITGAM**^**high**^ **subset**	High: *CCR2*, *ITGAM*, *CD14*	Undefined
	**CXCL8**^**high**^ **subset**	High: HIF1A, CXCL8	Undefined
	**IL10**^**high**^**TGFB1**^**high**^ **subset**	High: *IL10, TGFB1*	Immunosuppressive
**T**	**CD4**^**+**^ **naïve T cell**	High: *CD4*, *CCR7*, *LEF1*, *TCF7*, *SELL*	Dysfunctional
	**CD69**^**high**^**GZMA**^**−**^**CD4**^**+**^ **T cell**	High: *CD4*, *CD69*; Low: *GZMA*	Anti-tumor
	**CD69**^**high**^**GZMA**^**+**^**CD4**^**+**^ **T cell**	High: *CD4*, *CD69*, *GZMA*	Anti-tumor
	**CD4**^**+**^ **cytotoxic T**	High: *CD4*, *GZMA*, *GZMB*, *GZMH*, *GNLY*, *NKG7*	Anti-tumor
	**TH17-like cell**	High: *CD4*, *RORC*, *KLRB1*	Dysfunctional
	**Treg**	High: *CD4*, *IL2RA*, *FOXP3*; Low: *CD127*	Immunosuppressive
	**Proliferating T cell**	High: *CD4*, *MKI67*, *PCNA*, *HAVCR2*, *LAG3*, *PRDM1*, *TIGIT*, *CTLA4*, *TOX*	Dysfunctional
	**IFN-CD4**^**+**^ **T cell**	High: *CD4*, *IFIT3*, *ISG15*, *ISG20*, *MX1*, *IFNAR1*, *LAG3*, *PRDM1*, *TIGIT*	Dysfunctional
	**GZMA**^**low**^**GNLY**^**+**^**CD8**^**+**^ **T cell**	High: *CD8*, *GNLY*; Low: *GZMA*	Anti-tumor
	**TIGIT**^**+**^**CD8**^**+**^ **T cell**	High: *CD8*, *LAG3*, *TIGIT*, *GZMH*, *GZMA*, *GNLY*, *PRF1*, *GZMB*	Dysfunctional
	**Naïve CD8**^**+**^ **T**	High: *CD8*, *TCF7*, *SELL*, *LEF1*, *CCR7*	Undefined
	**GZMA**^**low**^**GNLY**^**low**^**CD8**^**+**^ **T cell**	High: *CD8*, *CD69*, *RUNX3*, *IL7R*; Low: *KLRG1*, *ITGAE*, *B3GAT1*, *GZMA*, *GZMB*, *GZMH*, *GZMK*, *GNLY*, *PRF1*, *NKG7*	Anti-tumor
	**GZMA**^**+**^**GNLY**^**low**^**CD8**^**+**^ **T**	High: *CD8*, *GZMA*, *TIGIT*, *PDCD1*, *CTLA4*	Dysfunctional
**NK**	**NK cell**	High: *TYROBP*, *KLRF1*, *FCGR3A*, *NCAM1*, *CD160*, *GZMA*, *GNLY*, *PRF1*, *GZMB*, *TIGIT*, *HAVCR2*; Low: *CD3D*, *CD3E*, *CD8A*, *CD8B*	Dysfunctional

CD1C^+^ DC (cluster 3) express *AREG*, *S100A8*, *S100A9*, *CD86*, and *CD1C* at high levels. And Cluster 4 was identified as CX3CR1^+^ DC with *CD8A* expression. CD1C^+^ DC shows the cDC1 gene expression pattern, while cDC2 pattern in Cluster 1–4, and pDC pattern in cluster 0 (Fig. 2c). cDC1s are critical for eliciting anti-tumor CD8^+^ T cell responses and T helper 1 (TH1) responses, cDC2 for CD4^+^ T cell response, and pDC for producing large amounts of type I IFN [40]. These results indicated that T cell response biased to CD4^+^ components, but not CD8^+^ components. The 5 clusters also show obvious differential expression pattern of transcription factor (Supplementary Figure 2B), pattern recognition receptors (Supplementary Figure 2C), cell adhesion/migration molecules (Supplementary Figure 2D), cytokines (Supplementary Figure 2E), and chemokine receptors (Supplementary Figure 2F).

As Fig. 2d shown, healthy donor BM-derived DC cells mainly located on the CD1C^+^ DC population. Compared with healthy states, AML states downregulated the expression of *SELL* (*CD62L*), *CD44*, and *CD2*, which indicates that the cell adhesion/migration function of AML patient BM-derived DC is changed (Supplementary Figure 2D). As known, DC plays critical roles in T cell response, so we illuminated the expression level of T cell function-related costimulatory and coinhibitory molecules (Fig. 2e). CD1C^+^ subset express many functional molecules, such as *CD80*, *CD86*, *VEGFA*, *CXCL8*, *CXCL2*, *IL1B*, *IL1RN*, *IL10*, *IL15*, *IL23A*, *IL7*, *OSM*, *VEGFB*, and *GDF9* (Fig. 2f). And found that CLEC7A^+^ DC cluster showed low levels of these molecules except *TNFSF9*, CX3CR1^+^ DC cluster with high levels of costimulatory and coinhibitory molecules, and other clusters with high levels of costimulatory molecules. CLEC7A is a functional receptor in DC/Macrophage to enhance NK cells-mediated tumoricidal activity [41]. And high expression of *TNFSF9* (*4-1BBL*) also indicated this population might enhance T cell-mediated tumoricidal activity, as previously reported [42], but significantly corrected with poor prognosis puzzlingly (Fig. 2g). CLEC7A^+^ DC appeared in a few AML samples (AML475.D0, AML371.D0, and AML556.D31). pDC subset express much more *TNFSF4*, *TNFSF13*, *LTB*, *HGF*, and *CCL28* (Fig. 2f), which appeared in many AML samples (AML1012.D0, AML210A.D0, AML329.D0, AML419A.D0, AML556.D31, AML707B.D41, and AML921A.D0). TNFSF4 (OX40L) provides co-stimulatory signals to enhance T cell function through TRAF2 and TRAF5 [43]. DC-derived TNFSF13 (APRIL) showed antitumor potential by upregulating proliferation and survival of T cells [44]. HGF is a negative cytokine of cancer immunotherapies involved in reactive recruitment of neutrophils, which can impair T cell expansion and effector function [45], but significantly corrected with good prognosis (Fig. 2g). And CCL18 is associated with recruitment of tumor-associated macrophages (TAMs), Treg, tumor-associated dendritic cells (TADCs), and cancer-associated fibroblast (CAFs). Even so, the direct costimulation of TNFSF4 and TNFSF13 is the mainstream power to drive the antitumor potential. FCGR2A (CD32a) and FCGR2C (CD32c) are critical for phagocytosis and cross-presentation of antibody-coated antigens, but FCGR2B (CD32b) as an inhibitory FcγR [46]. CD206^+^ DC subset with high expression of both activate and inhibitory FcγR, might involve a specific dysfunction of immune checkpoint. We also found *TNFSF12*, *TNFSF8* (*CD30L*), and *IL18*, expressed at high levels in CD206^+^ DC cells (Fig. 2g). TNFSF8 plays both positive and negative roles in T cell-mediated immune function [43], but it has more important roles in regulating Treg function under AML status based on the high CD30 expression level in Treg and dysfunctional proliferation T subset (Fig. 4d). IL18 combining different cytokines can induce different immune responses, such as type 1 response, type 2 response, and innate-type allergic inflammation [47]. *IL16*, *IL32*, *CCL5*, *CCL23*, *PDGFA*, *PDGFC*, *TNFSF14*, *TNFSF10*, *XCL1* and *XCL2* were highly enriched in the CX3CR1^+^ DC subset. TNFSF10 (TRAIL), as a proapoptotic ligand, involved the CD4^+^ T cell apoptosis mediating by TRAIL/DR5 signaling [48]. Proinflammatory TNFSF14 (LIGHT) expresses lower than TNFSF10. And PDGF was expressed by TADC [49], involved formation of tumor microenvironment [50] and Treg induction [51]. The proportions of this CX3CR1^+^ DC subset are increased in most AML samples (AML1012.D0, AML210A.D0, AML329.D0, AML329.D20, AML419A.D0, AML707B.D41, and AML707B.D112). The CX3CR1^+^ DC subset expressed *CD274* and *PDCD1LG2*, as the ligands of PDCD1 mediating T-cell suppression. Taken together, immunosuppression-related DC cells increased in most AML samples, especially Treg-related CD206^+^ DC and T cell suppression-related CX3CR1^+^ DC, which infirmed by the survival analysis of TCGA AML data (Fig. 2g and Supplementary Figure 2F). And expression of several DC-related genes (*TGFB1*, *MRC1*, *CLEC7A*, *ITGAX*, *ITGB2*, *CX3CR1*, *CCL22* and *TNFSF8*) significantly corrected with poor prognosis of TCGA AML patients (Fig. 2g).

Dysfunctional DC impairs innate and adaptive immune response. Educated DC, as mentor of T/NK cells, has immense potential in tumor immunotherapy [52]. Our results showed that AML patient-derived DC compositions changed whatever treated or not. And two immunosuppressive DC subset, CD206^+^ DC and CX3CR1^+^ DC became the mainstream, which might influence immunotherapy. These results note us to identify the major DC subset, before adopting specific DC-based tumor immunotherapy. Several clinical studies based on DC vaccines showed favorable outcomes in AML patients [53, 54]. But it just worked in some patients, which might remind us that the subsets and states of AML patient autologous DC influenced the DC-based tumor immunotherapy. We can harvest more powerful antitumor-DC by metabolic status reprogramming [55] and DC regeneration from induced pluripotent stem cell (iPSC)/other somatic cells [56, 57].

### The diverse immune phenotypes of macrophages in different AML patients

**Fig. 3:**
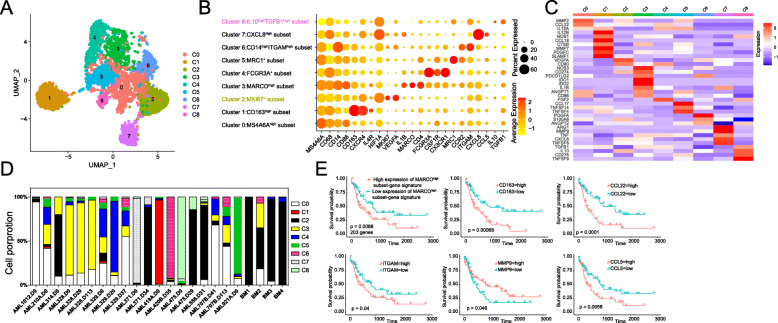
Dissection and clustering of mature myeloid lineages in AML patients and healthy donors. **a** UMAP plot of Monocyte/Macrophages from Fig. 1a-represented Mono/Mac cluster. These mature myeloid cells can be divided into 9 subsets without CD14^+^CD3D^+^ subset. **b** Dot plot of differentially key cell-type marker genes. **c** Heatmap of mean expression of selected cytokine genes in each cell subset. **d** Proportion of 9 mature myeloid subsets in myeloid population subsets in each AML patient or healthy donor. Only samples of containing ≥20 myeloid cells were represented. **e** The Kaplan-Meier overall survival curves of TCGA AML patients grouped by specific MACRO^+^ subset gene sets and myeloid-related genes (*CD163*, *ITGAM*, *MMP9*, and *CCL5*). + represents censored observations, and *P* value was calculated by multivariate Cox regression

In order to better understand the heterogeneity of monocytes and macrophages within and across AML patients and healthy donors, we extracted and clustered this population (4487 cells) by UMAP (Supplementary Figure 3A), and got 10 subsets (Cluster 0:MS4A6A^high^ subset, Cluster 1:CD163^high^ subset, Cluster 2:MKI67^+^ subset, Cluster 3:MARCO^high^ subset, Cluster 4:FCGR3A^+^ subset, Cluster 5:MRC1^+^ subset, Cluster 6:CD14^high^ITGAM^high^ subset, Cluster 7:CXCL8^high^ subset, Cluster 8:IL10^high^TGFB1^high^ subset, and Cluster 9:IFNG^high^ subset) (Table 1). Cluster 9:IFNG^high^ subset (119 cells) express both *CD14* and *CD3D* (Supplementary Figure 3B), consisted of T cells bound to monocytes or monocyte debris [58], and were excluded in subsequent analysis (Fig. 3a).

Interestingly, MKI67^+^ subset is the major composition with *VEGFA*-expressing at high level in healthy donors BM-derived monocytes/macrophages (Fig. 3b). MS4A6A^high^ subset is a common population in most AML samples and part of normal healthy donor samples. This subset showed an M2-like pattern (*CSF1R*, and *TGFB1*) and expressed a Treg-attracting chemokine *CCL22* (Fig. 3c). CD163^high^ subset with M2 phenotype (*CD163*, *CXCR4*, *IL4R*, *TGFB1*, and *CCL18*) is a novel population, and as a dominant population in AML419 but not other patients. *CX3CR1* and *CSF1R* are highly expressed in FCGR3A^+^ (CD16^+^) subset, which can be identified as an inflammatory phenotype in anti-tumor immune response [59]. Although in preclinical models of glioblastoma, targeting TAMs and microglia using CSF1R inhibitor combined with radiotherapy could enhance survival [60], might be not suitable for AML patients and impairs anti-tumor immune response [61]. This reminds us CSF1R inhibitors (such as BLZ945 and PLX3397) might not bring benefit to some AML patients (AML210A, AML329, and AML707B) without identifying major groups of CSF1R-expressing immune cells. In solid tumor models (melanoma, breast cancer, and colon carcinoma), pattern recognition scavenger receptor MARCO defined immunosuppressive TAMs. And antibody targeting this subset can enhance the effect of anti-CTLA4 checkpoint therapy to block the tumor growth and metastasis [62]. Cluster 3:MARCO^high^ subset showed higher expression of several typic genes (M1-like gene: *IL1B*; M2-like genes: *CD163*, *IL4R*, *CSF1R*, *CX3CR1*,and *TGFB1*) than MKI67^+^ subset, which was positively correlated with M2-like phenotype. Anti-MARCO immunotherapy might especially benefit AML patient (AML210A, AML314, AML328, AML329, and AML707B) with high frequencies of MARCO^high^ subset. MRC1^+^ subset also showed a M2-like phenotype with high expression of *MRC1* (*CD206*), *CSF1R*, and *TGFB1*, and enriched in some AML patients, such as AML210A, AML707B, and AML921. And these patients might benefit from innate defense regulators RP-182, which can trigger a conformational switch of CD206 and enforce the TAMs to M1-like cells [63]. CXCL8^high^ subset with high expression of *HIF1A*, also expressed other important cytokines or regulators, such as *MMP9*, *TNF*, *IL1B*, *VEGFA*, *CCL5*, *TNFSF8*, and only enriched in AML371 patient (Fig. 5d). For AML475, chemotherapy can decrease the frequency of a high immunosuppressive IL10^high^TGFB1^high^ populations (Fig. 3d). What’s more, MARCO^high^ subset-gene signature and expression of several macrophages-related genes (*CD163*, *ITGAM* and *CCL5*) significantly corrected with poor prognosis of TCGA AML patients, but MMP9 with good prognosis (Fig. 3e and Supplementary Figure 3C).

Many macrophage-based therapies had been developed, such as anti-SIRPα antibody [64], anti-MARCO antibody [62], chimeric antigen receptor macrophages (CAR-M) [65–67], and adoptively transferred macrophages with IFN-γ backpacks [68]. Mono/Mac compositions of AML patients showed substantial variation. The large proportional differences of Mono/Mac subsets indicated the immune status are diverse in different AML patients. This might note us simplex immunotherapy, by driving the directed differentiation of Mono/Mac, is limited and variable for AML treatment.

### Burnt conventional T/NK lineage and expanding suppressive subsets in AML BM microenvironment

**Fig. 4:**
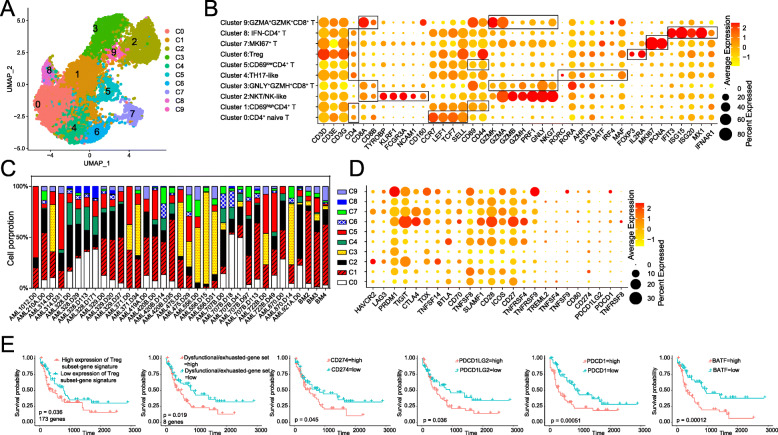
Diversity of T/NK subsets revealed by scRNA-seq analysis. **a** UMAP plot of sc-RNAseq data (*n* = 10,096 cells) showed 10 distinct clusters. **b** Dot plot of differently key cell-type marker genes. **c** Histogram showed the fractions of different cell-type in T/NK populations for each AML patient and healthy donors’ BM cells, colored based on cell type. **d** Dot plot showed the transcript expression pattern of stimulation molecules and their receptors. **e** The Kaplan-Meier overall survival curves of TCGA AML patients grouped by specific Treg gene sets, dysfunctional/exhausted-gene set (*LAG3*, *TIGIT*, *CTLA4*, *HAVCR2*, *TOX*, *PDCD1*, *CD274*, *PDCD1LG2*), and several genes (*CD274*, *PDCD1LG2*, and *BATF*). + represents censored observations, and *P* value was calculated by multivariate Cox regression

To comprehensively analyze the functional change of T/NK lineage in AML states, we utilized UMAP for identifying T subsets with more details (Fig. 4a). Thus, the T/NK lineage was divided into 10 clusters (Table 1). CD4^+^ naïve T cells (Cluster 0) was identified by the high expression of naïve/TCM-state-related genes (*CCR7*, *LEF1*, *TCF7*, and *SELL*) (Fig. 4b). CD69^high^ CD4^+^ T cells (Cluster 1) was identified by high expressing *CD69*, and CD69^low^ CD4^+^ T cells (Cluster 5) accordingly. Of note, Cluster 4 (dubbed “TH17-like cells”) express *RORC*, *RORA*, *STAT3*,*BATF*, *AHR*, *IRF4*, and *MAF*. Cluster 6 (dubbed “Treg cells”) uniquely express *IL2RA* and *FOXP3* at high levels; Proliferating T cell cluster (Cluster 7) express canonical proliferation markers *MKI67* and *PCNA*. IFN-CD4^+^ subpopulation (Cluster 8) express interferon-stimulated genes (ISGs) (*IFIT3*, *ISG15*, *ISG20*, *MX1*, and *IFNAR1*). CD8^+^-T/NK/NKT cells comprised 3 clusters distinct from CD4^+^ T cells and included: NK/NKT-like cells (Cluster 2) expressing cytotoxic genes (*GZMA*, *PRF1*, *GNLY*, *NKG7*, *GZMB*, and *GZMH*) and NK-related genes (*TYROBP*, *KLRF1*, *FCGR3A*, *NCAM1* (*CD56*), and *CD160*); Cluster 3 (dubbed “GNLY^+^GZMH^+^CD8^+^ cells”) expressing *CCL5*, *GNLY*, and *GZMH* at high levels; Cluster 9 (dubbed “GZMK^+^GZMA^+^CD8^+^ cells”) expressing *CD160*, *GZMK*, and *GZMA*.

To uncover the T/NK functional states in AML state before and after treatment, we assessed the proportion of different subsets to total T/NK lymphocyte in both AML patients and healthy individuals. The proportions of CD4^+^ naïve T cells to total T/NK lymphocytes in most AML patients are much more than healthy individuals, whatever before or after treatment (Fig. 4c). Moreover, chemotherapy could promote the terminal differentiation of naïve T helper cells except AML328 and AML722B. The results profiled that AML-states inhibited the terminal differentiation of CD4^+^ naïve T cells, and eliminating AML cells can reverse this state in most cases, which was reported by other researchers [32]. CD69^high^CD4^+^ T cells accounted for 60% of T/NK lymphocytes in the healthy individuals’ BM. Only parts of AML patients (such as AML707B and AML420B) showed the increasing proportions of CD69^high^CD4^+^ T cells after treatment obviously. The chemotherapy cannot drive the naïve T helpers towards to CD69^high^CD4^+^ activating T subset in most cases. And CD69^low^CD4^+^ T cells appeared in some samples (such as AML1012.D0, AML314, AML722B, and AML371) at high frequencies, which indicated these CD4^+^ T cells cannot be activated for performing their immune-activated function. The proportions of TH17-like cells to total T/NK lymphocytes in most AML patients are much more than healthy individuals, whatever before or after treatment (Fig. 4c, Supplementary Figure 4A), which is positively correlated with increasing IL6 secreted from malignant cells [7]. As shown, TH17-like T cells is rare in BM cells of healthy individuals. On the previous reports, TH17 cells can be regarded as a more favorable outcome in AML patients, controversially [69, 70]. We then analyzed the proportion of Treg cells by total T/NK lymphocytes in AML patients. Parts of AML samples, but not all, showed significantly higher levels of Treg subpopulation. Interestingly, the proportions of Treg cells increased within 4 weeks after treatment and then decreased. The increased Treg cells might create an immunosuppressive niche and impair the immune activation for eliminating malignant cells. And this indicated that immunotherapy of inhibiting Treg expansion and function within 4 weeks is the most effective and economical treatment strategy to enhance the treatment effect of conventional chemotherapy. Interestingly, some AML samples (AML556, AML420B, AML475, and AML707B) showed higher proportions of proliferation T cell subsets. As previously reported, dysfunctional T cells are the major intratumoral proliferating T cell compartment with dysfunctional signature [71]. Although the proliferation T cluster showed the proliferation-related markers (*MKI67* and *PCNA*), this cluster acquired a dysfunctional signature of expressing *LAG3*, *TIGIT*, *CTLA4*, *HAVCR2*, and *TOX* at high levels, similar to the immunosuppressive signature of Treg. We observed a strong ISGs pattern in cluster 8 in AML patients (AML328, AML329, and AML707B). The IFN response module can mark the activated CD4^+^ T cells, but acquired a dysfunctional signature of expressing *LAG3*, *PRDM1*, and *TIGIT*.

AML samples showed higher proportions of GNLY^+^GZMH^+^CD8^+^ T cells and lower proportions of GZMK^+^GZMA^+^CD8^+^ T cells compared to healthy donors. Interestingly, NKT/NK-like cluster and GZMK^+^GZMA^+^CD8^+^ T cluster showed significant exhaustion-related genes (*LAG3*, *TIGIT*, *HAVCR2*, and *TOX* in NKT/NK-like cluster; *PDCD1*, *LAG3*, *TIGIT*, *CTLA4*, and *TOX* in GZMK^+^GZMA^+^CD8^+^ T cluster) (Fig. 4d) identified in many kinds of tumors [72, 73].

In conclusion, under the AML states, T/NK cells showed a diversity of subpopulations and function, and transited into immunosuppressive states. Our analysis of TCGA AML data showed the Treg and dysfunctional/exhausted T/NK subsets might represent poor prognosis (Fig. 4e and Supplementary Figure 4B). Immunotherapies have changed the methods of cancer treatment subversively, such as the application of checkpoint inhibitors targeting PD-1 and CTLA-4 [74, 75]. But these strategies are not always efficacious, which is partly due to the difference of heterogeneity and/or ability of tumor-infiltrating T lymphocytes. Our results identified distinct states of T/NK cell lineages, which confirmed that different response of checkpoint inhibitors in different AML patients with different outcomes. Importantly, increasing frequencies of TH17-like cluster and Treg cluster, as common cell signature, are obvious in AML patients. And the dynamic changes of T/NK subsets also might be associated with the outcomes, and help to adjust the immunotherapies.

### Novel T clusters and functional states in AML patients

**Fig. 5:**
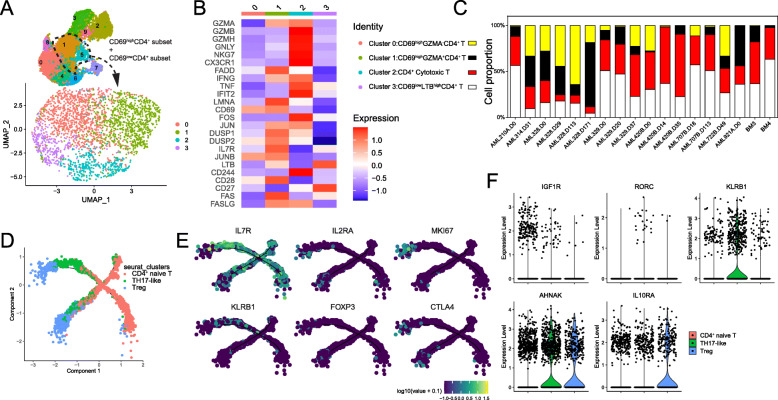
Unique CD4^+^ subsets revealed by scRNA-seq analysis. **a** UMAP plot of CD69^high^CD4^+^ population and CD69^low^CD4^+^ population. These 2515 cells can be divided into 4 subsets. **b** Heatmap showing average expression level of cell-type genes expressed by the 4 clusters. **c** Histogram showed the fractions of different cell-type in CD69^high^CD4^+^ population and CD69^low^CD4^+^ population for each AML patient and healthy donors’ BM cells, colored based on cell type. **d** The branched trajectory of state transition of naïve CD4^+^ T cells, TH17-like cells, and Treg cells in a two-dimensional state-space inferred by Monocle (version 2.14.0). Each dot corresponded to one single cell, colored according to its cluster label. **e** Expression maps showing log-normalized expression of typical markers (*IL7R*, *IL2RA*, *MKI67*, *KLRB1*, *FOXP3*, and *CTLA4*) in the differentiation of Naïve CD4^+^ T to TH17-like cells and/or Treg cells. Data are shown as log-normalized expression. Yellow indicates high expression, dark blue indicates low expression. **f** Violin plot showing the expression levels of functional genes (*IGF1R*, *RORC*, *KLRB1*, *AHNAK*, and *IL10RA*)

To characterize the T/NK cellular diversity in AML BM microenvironment, we further analyzed specific clusters for detecting more function and state changes. We acquired the expression data of CD69^high^CD4^+^ T population and CD69^low^CD4^+^ T population for UMAP (Fig. 5a), and found a unique cytotoxic CD4^+^ T subset existed in both AML patients and healthy donors, and CD69^low^ LTB^high^CD4^+^ T subset in AML patients but rarely in healthy donors (Fig. 5b and Fig. 5c) (Table 1). Cytotoxic CD4^+^ effectors, with anti-tumor activity, was identified by other researchers in other tumors [76–78], and might lead to therapeutic benefit. And we first identified a cytotoxic CD4^+^ T subset under AML stress by scRNA-seq analysis. Although CD69^low^LTB^high^CD4^+^ T subset is approximated T cell: erythrocyte complexes (Supplementary Figure 5A), which might involve cell:cell communication events, but not technical artefact or random association [58].

As known, TH17 lineage and Treg lineage strongly linked, and differentiation imbalance leads to abnormal immune states [79]. So we then analyzed the developmental trace and function of naïve CD4^+^ T cells, TH17-like cells, and Treg cells (Fig. 5d). The expression of signature genes and known functional markers suggested the direction of naïve CD4^+^ T population to TH17-like population, nearly TH17/Treg intermediate population, and then terminal Treg population (Supplementary Figure 5B). These 3 populations have different functional gene expression pattern, such as high expression level of *IGF1R* in Naïve CD4^+^ T cell, *RORC* and *KLRB1* in TH17/Treg intermediate population, and *IL10RA* in Treg population (Fig. 5e, Supplementary Figure 5C). Signaling of IGF-IGF1R drive the favorite of naïve T to TH17 but not Treg, and prime the TH17 cell fate [80]. KLRB1 is a remarkable proinflammatory marker of TH17 cells and proinflammatory FoxP3^+^ cells [81, 82]. RORC, as a faithfully TH17-specific transcript factor, balances the generation of TH17 and Treg subsets, and expresses at TH0-like intermediate population toward to TH17 subset and Treg subset [83]. Triggered signaling of IL10RA is important for Treg-mediated suppression of TH17 proinflammatory response [84]. To illustrate the state transition of these 3 populations, we performed branched trajectory analysis by Monocle (Fig. 5d). As shown in Fig. 5d, TH17/Treg intermediate population is halfway between naïve CD4^+^ T cells and Treg cells. As expected, Treg-specific genes (*IL2RA*, *FOXP3*, and *CTLA4*) express at high levels in left terminal branches, which enriched Treg cells. And *KLRB1* express at high level on the left side of the above branch. *IL7R* can be used as a marker to exclude non-Treg cells, which located on the non-Treg positions. Interestingly, Treg population was divided into MKI67^+^ activated Treg subset and MKI67^−^ resting Treg subset. In the healthy donor, resting Treg cells and naïve CD4^+^ T cells are the major population in state 1 and 6. But in the AML state, Naïve CD4^+^, TH17/Treg intermediate cells, and MKI67^+^ Treg cells are increasing before and after treatment (Supplementary Figure 5D). This reminds us that arresting the directed differentiation of TH17/Treg intermediate cells to Treg cells, or transdifferentiation of TH17 to Treg, might be beneficial for therapeutic effect combing conventional chemotherapy.

**Fig. 6:**
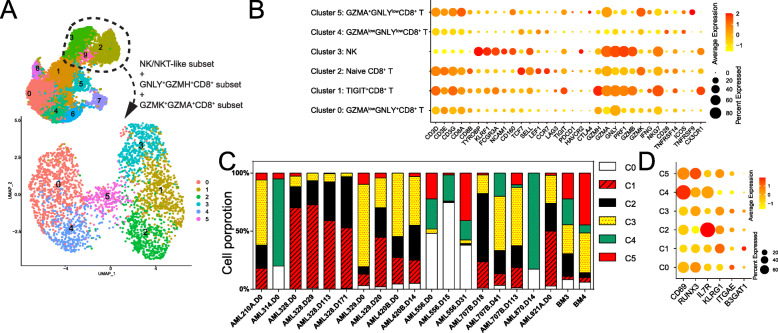
Dysfunctional/exhausted CD8^+^-T/NK subsets revealed by scRNA-seq analysis. **a** UMAP plot of NK/NKT-like population, GNLY^+^GZMH^+^CD8^+^ T population, and GZMK^+^GZMA^+^CD8^+^ T population. These 2901 cells can be divided into 6 subsets. **b** Dot plot showed the transcript expression pattern of cell-type genes. **c** Histogram showed the fractions of different cell-type in NK/NKT-like population, GNLY^+^GZMH^+^CD8^+^ T population, and GZMK^+^GZMA^+^CD8^+^ T population for each AML patient and healthy donors’ BM cells, colored based on cell type. **d** Dot plot showed the transcript expression pattern of memory-like CD8^+^ T-related genes

To feature the changes of CD8^+^-T/NKT/NK cells, we analyzed three related populations (NK/NKT-like cluster, GNLY^+^GZMH^+^CD8^+^ cluster, and GZMK^+^GZMA^+^CD8^+^ cluster) by UMAP method with more details (Fig. 6a). These clusters can be divided into 6 small subsets (Cluster 0:GZMA^low^GNLY^+^CD8^+^ subset, Cluster 1:TIGIT^+^CD8^+^ T subset, Cluster 2:Naïve CD8^+^ T subset, Cluster 3:NK subset, Cluster 4:GZMA^low^GNLY^low^CD8^+^ T subset, and Cluster 5:GZMA^+^GNLY^low^CD8^+^ T subset) (Fig. 6b) (Table 1). Interestingly, the proportions of GZMA^+^GNLY^low^CD8^+^ T subset to total CD8^+^-T/NKT/NK in most AML patients, were decreased. And the GZMA^+^GNLY^low^CD8^+^ T subset showed exhausting gene expression pattern (*TIGIT*, *PDCD1*, and *CTLA4*). Meanwhile, TIGIT^+^CD8^+^ T population was increased in most AML patients obviously, and GZMA^low^GNLY^+^CD8^+^ T population in part of AML patients (AML556) (Fig. 6c). TIGIT is an inhibitory receptor expressed on dysfunctional T cells, as previously reported [85]. And blockade of TIGIT targets CD8^+^ CTL or NK cells, prevents exhaustion and promotes target [85, 86]. TIGIT-blocking strategies might enhance the AML treatment mediated by CD8^+^ CTL and NK cells in most cases (except AML314.D0, AML556.D15, and AML870.D14). Likewise, blockade of PDCD1 and CTLA4 also enhance the AML treatment. GZMA^low^GNLY^low^CD8^+^ T subset expressed low levels of granzyme genes (*GZMA*, *GZMB*, *GZMH*, and *GZMK*), *GNLY*, *KLRG1*, *ITGAE*, *B3GAT1*, and *PRF1*, but high levels of *TCF7*, *RUNX3*, *CD69*, and *IL7R* (Fig. 6b and d), which is consisted with phenotype of CD8^+^ memory T cells [87, 88]. This subset plays positive roles in improving cancer immunotherapy [89]. This CD8^+^ memory-like cells have a high proportion in part of samples (AML314.D0, AML556.D0, AML556.D15, AML556.D31, AML707B.D41, and AML870.D14). In most AML patients, the proportion of transitional GZMA^+^GNLY^low^GZMK^+^CD8^+^ effector T subset (except AML556.D0, AML556.D31,) and GZMA^low^GNLY^+^ cytotoxic T subset (AML314.D0, AML556.D0, AML556.D15, AML556.D31, and AML870.D14), are decreased. And naïve-like CD8^+^ T subset with inhibitory molecules pattern (*LAG3*, *TIGIT*, and *CTLA4*), seems to be repulsive to CD8^+^ memory-like subset.

Detailed classification of tumor-infiltrating lymphocytes (TILs) and identification of cellular crosstalk in AML BM niche will provide important means of assistance based on existed mature chemotherapies [90]. By examining immune cell types can help to formulate personalized high-efficiency immunotherapy trials. Our results showed AML-derived TILs exhibited heterogenous, and combined immunotherapy strategies based on heterogenous cell-types (such as Treg, TH17-like cells, exhausted NK cell, dysfunctional CTLs, and cytotoxic CD4^+^ T cells) provide assistance for inhibiting relapse after chemotherapy.

## Discussion

Macrophages is a plastic heterogeneous population, which involved the survival and drug resistance of AML. Neonatal macrophages can mature into classically activated (M1) and alternatively activated (M2) macrophages depending on the microenvironment. Meanwhile, M1 macrophages and M2 macrophages can translate into each other with changes of immune status. Repolarization of M2 macrophages toward M1 macrophages is helpful to establish new therapeutic targets in AML [91]. Our results showed the diversity of macrophages in AML BM niche. As reported in Renca tumor, single anti-CSF1R treatment decreased part of TAMs, which suggested differential sensitivity of distinct macrophage subsets for specifical antibody-drug [92]. But anti-CSF1R treatment for AML should consider the major CSF1R-expressing population in BM microenvironment, and avoid to eliminate anti-tumor population and impair T cell response [61]. *Yu* and his colleagues also showed us anti-CD40 agonist therapy could amplify immune-activated cDC1 subset, increase effector memory CD8^+^ CTLs, and induce the activation and expansion of TH1-like CD4^+^ T cells in MC38 tumor model [92]. Targeting mature myeloid lineages is an attractive therapeutic approach for AML, but firstly need to identify the mainstream of myeloid subset in BM microenvironment. The scRNA-seq analysis can help to identify the tumor microenvironment information in detail, and implement more effective immunotherapy approaches combined with conventional chemotherapy.

Many animal models showed us the evidence of antileukemic T-cell immunity with exhausted program or antigen-specific T cell tolerance [93, 94]. Our results identified the abnormal T-cell subset alterations in AML before and after treatment by scRNA-seq analysis. Augmented Treg is an obvious feature of newly diagnosed AML patients, and the proportion of Treg cells increased within 4 weeks after treatment, which are at odds with other research based on other research approaches [95–98]. Tumor microenvironment components secrete immunosuppressive chemokines, such as CCL17 and CCL22, to attract Treg cells. And dysfunctional DC populations expand Treg population mediated by cytokines and costimulatory [99]. As previously reported, activated Treg, with antigen stimulation and oligoclonal skewing, presented during early lymphocyte recovery, which is consistent with our results of scRNA-seq analysis. Furthermore, our data also indicated that there exist two Treg subsets with different states, function, or development path, in AML BM microenvironment. Meanwhile, Treg cells not only impairs conventional T-cell function, but also induces DC apoptosis and dysfunction [99]. We also found several dysfunctional DC subsets involved in the AML process, especially CX3CR1^+^ DC subset with high expression level of *CD274* and *PDCD1LG2*. These dysfunctional DC subsets, conspire with Treg cells, contributed to the T cell exhaustion and anergy. And increased TH17-like cells, as an abnormal population in BM microenvironment, closed to a TH17/Treg intermediate state, might illustrate the contradictory of TH17 phenotype and function in AML patients [100, 101]. A tumor-bearing mouse model showed tumor-infiltrating Treg cells can be converted from IL17A^+^FoxP3^neg^ cells fostered by TGFβ and PGE2 [102], which is consistent with our results about two Treg states and development pathways of naïve CD4^+^ T cell to TH17-like subsets and then Treg subsets in AML patients.

## Conclusions

In summary, we analyzed scRNA-seq dataset of AML patient-derived BM cells and characterized their immune cell landscape with more details (Table 1). What’s more, these results can help us to predict the prognosis of AML (Table 2). We also identified several infrequently reported immune cell types in AML patients, such as TH17/Treg intermediate population, CD8^+^ memory T cells, different types of macrophages, and dysfunctional DC subsets. Exhausted conventional T cells and immunosuppressive T cells (Treg and other T subsets) can be used as targets of anti-CTLA4, anti-PD1, and anti-CD25 therapies. But these don’t always work because of the diversity of T/NK cells and other immune cell types. So the explosive emergence of immune-regulated drugs emphasizes the strong need for identification of predictive biomarkers, which help to illustrate which cell populations are the most critical targets in AML. Additionally, inhibition of TH17/Treg intermediate cells toward to Treg direction should be noted, which can be developed as a new immunotherapy strategy. What’s more, we found that mature myeloid lineages exist extremely high diversity of monocyte/macrophages and DC. Targeting single or small macrophage subset and/or DC subset, don’t seem to work for most AML patients. But as our scRNA-seq analysis of AML BM cells, this approach might be as a mean of diagnosis to help identification of effective immunotherapy strategies by targeting macrophages, DC, and TILs [103]. With the maturation and popularization of scRNA-seq technology, this technology will provide more and more details about occurrence of disease, selection and development of treatment and prognosis approaches, and predicting disease risk, as it is used in SARS-CoV-2 prevention and treatment [104–106].

**Table 2:** Summarization of major immune cell subset in AML BM microenvironment and prognosis

Cluster	Subset	Representative prognostic genes	Prognosis
**Mast cell**	**–**	Mast cell-gene signature (215 genes)	Good
**DC**	**CD206**^**+**^ **DC subset**	CD206^+^ DC-gene signature (111 genes), *MRC1*, *TNFSF8*	Poor
	**–**	*CX3CR1*, *TGFB1*, *CLEC7A*, *ITGAX*, *ITGB2*	Poor
**Mono/Mac**	**MARCO**^**high**^ **subset**	MARCO^high^ subset-gene signature (203 genes)	Poor
	**–**	*CCL22*, *CD163*, *ITGAM*, *CCL5*	Poor
	**–**	*MMP9*	Good
**T**	**Treg**	Treg subset-gene signature (173 genes)	Poor
	**Dysfunctional/exhausted T**	Dysfunctional/exhausted-gene set (*LAG3*, *TIGIT*, *CTLA4*, *HAVCR2*, *TOX*, *PDCD1*, *CD274*, *PDCD1LG2*)	Poor
	**–**	*CD274*, *PDCD1LG2*, *PDCD1*, *BATF*	Poor

## Supplementary Information

**Additional file 1: Supplementary Table.**.

**Additional file 2: Supplementary Figure 1.** Analysis of differences in gene expression between BM-derived cells in AML patients and healthy donors, dynamic changes of cell-type proportion, and survival curves of TCGA AML patients. Expression of Hallmark signatures: top genes **(A)**, lineage marker genes **(B)**, HSPC pattern genes **(C)** and monocyte pattern genes **(D)**. **E,** dynamic changes of cell-type (CD8^+^-T/NK/NK, MKI67^+^ T, LYZ^hi^EREG^hi^ monocyte precursor, Mono/Mac, Erythroid lineage 1, Erythroid lineage 2, B cell, and Mast cell) proportion before and after treatment, and healthy donor-derived BM cells are represented at the end of plots. **F,** The Kaplan-Meier overall survival curves of TCGA AML patients grouped by the cluster-specific gene sets. + represents censored observations, and *P* value was calculated by multivariate Cox regression.

**Additional file 3: Supplementary Figure 2.** Dot plot of differentially surface markers **(A)**, transcription factors **(B)**, pattern recognition receptors **(C)**, cell adhesion/migration molecules **(D)**, and chemokine receptors **(E)**. **F,** The Kaplan-Meier overall survival curves of TCGA AML patients grouped by specific DC subset (pDC, CLEC7A^+^ DC, and CD1C^+^ DC) gene sets. + represents censored observations, and *P* value was calculated by multivariate Cox regression.

**Additional file 4: Supplementary Figure 3. A,** UMAP plot of Monocyte/Macrophages from Fig. 1a-represented Mono/Mac cluster. These mature myeloid cells can be divided into 10 subsets before filtering possible cell-cell complexes. **B,** Expression levels of CD14 and CD3D across Mono/Mac population illustrated in UMAP plots. **C,** The Kaplan-Meier overall survival curves of TCGA AML patients grouped by specific subset gene sets. + represents censored observations, and *P* value was calculated by multivariate Cox regression.

**Additional file 5: Supplementary Figure 4. A,** dynamic changes of proportion of distinct cell-types in total T/NK cells before and after treatment, and healthy donor-derived BM cells, as control, are represented at the end of plots. **B,** The Kaplan-Meier overall survival curves of TCGA AML patients grouped by specific NK/NKT-like gene set and IFN-CD4^+^ gene set. + represents censored observations, and *P* value was calculated by multivariate Cox regression.

**Additional file 6: Supplementary Figure 5. A,** Violin plot showing the expression levels of *HBA2*, *HBB*, and *LTB* in 4 clusters (CD69^high^GZMA^-^CD4^+^ T, CD69^high^GZMA^+^CD4^+^ T, CD4^+^ Cytotoxic T, CD69^low^LTB^high^CD4^+^ T) from Fig. 5a-represented cells. **B,** The state-space of Naïve CD4^+^ T cluster, TH17-like cluster, and Treg cluster. Each dot corresponded to one single cell, colored according to its state (total 6 states). **C,** Expression maps showing log-normalized expression of typical markers (*ITGB1*, *GZMA*, *IGF1R*, and *IL10RA*) in the differentiation of Naïve CD4^+^ T to TH17-like cells and/or Treg cells. Data are shown as log-normalized expression. Yellow indicates high expression, dark blue indicates low expression. **D,** Typic state-space of some AML samples (AML420B.D0 and AML420B.D14; AML328.D0, AML328.D29, AML328.D113, and AML328.D171) are represented, and BM4 as healthy control. Each dot corresponded to one single cell, colored according to its state (total 6 states).

## Data Availability

All data generated or analyzed in this study are included in this article. Other data that are relevant to this article are available from the corresponding author upon reasonable request.

## Abbreviations

AML: Acute myeloid leukemia
scRNA-seq: single-cell RNA sequencing
BM: Bone marrow
DC: Dendritic Cells
Treg: Regulatory T
TH17: T helper cell 17
ROS: Reactive oxygen species
IDO: Indolamine-2,3-dioxygenase
NK: Natural killer
MDSCs: Myeloid-derived suppressor cells
UMAP: Uniform manifold approximation and projection
HSPCs: Hematopoietic stem/progenitor cells
LSPC: Leukemia stem/progenitor cells
PB: Periphery blood
pDC: plasmacytoid dendritic cells
TAMs: Tumor-associated macrophages
TADCs: Tumor-associated dendritic cells
CAFs: Cancer-associated fibroblast
iPSC: induced pluripotent stem cell
CAR-M: Chimeric antigen receptor macrophages
