# Supplementary figures and images for: Single-cell map of diverse immune phenotypes in the acute myeloid leukemia microenvironment
Source: Biomark Res. 2021 Mar 1;9:15. doi: 10.1186/s40364-021-00265-0 (PMC7919996; doi:10.1186/s40364-021-00265-0)

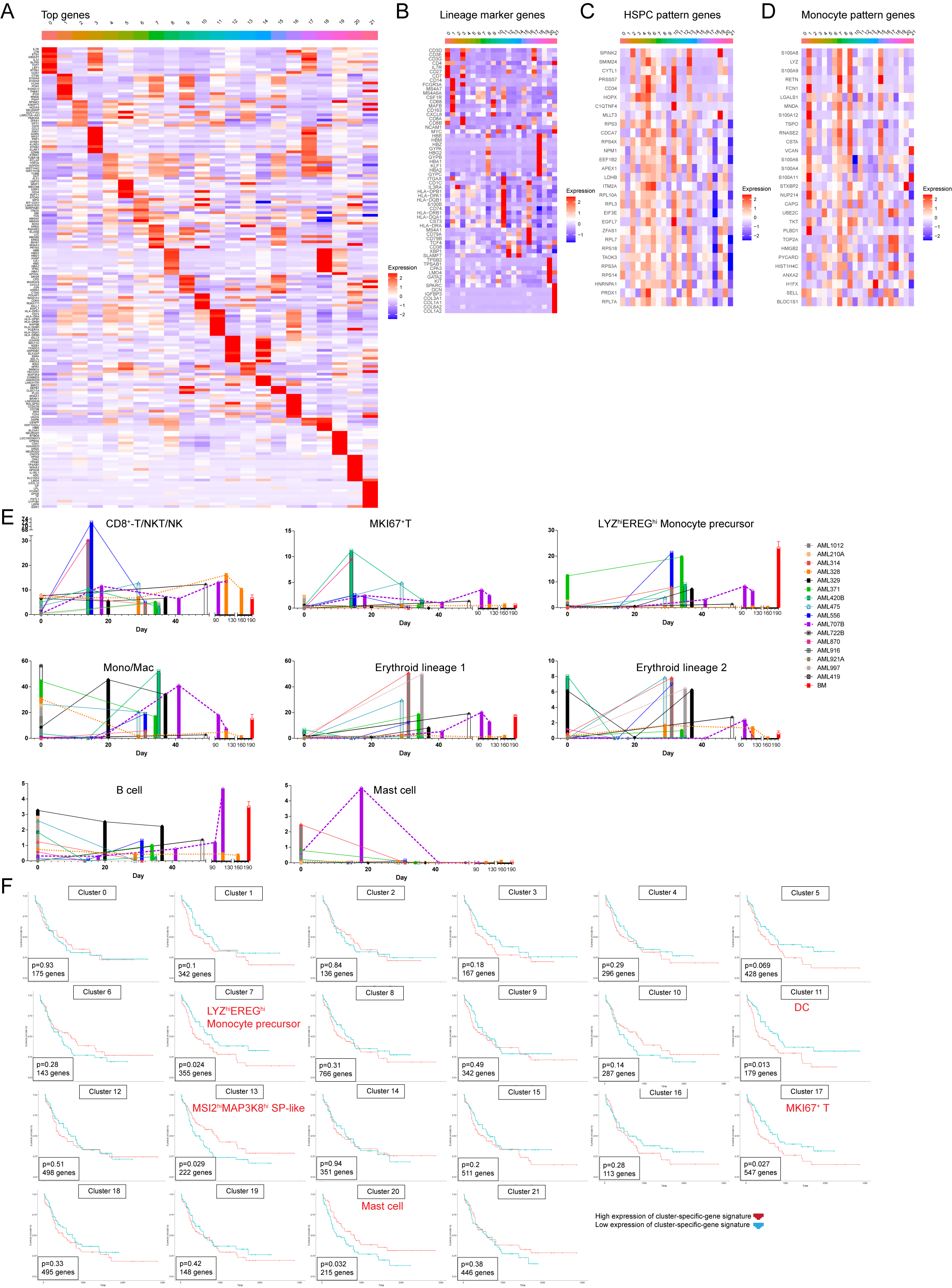

Supplement: Supplementary file 2 — Additional file 2: Supplementary Figure 1. Analysis of differences in gene expression between BM-derived cells in AML patients and healthy donors, dynamic changes of cell-type proportion, and survival curves of TCGA AML patients. Expression of Hallmark signatures: top genes (A), lineage marker genes (B), HSPC pattern genes (C) and monocyte pattern genes (D). E, dynamic changes of cell-type (CD8+-T/NK/NK, MKI67+ T, LYZhiEREGhi monocyte precursor, Mono/Mac, Erythroid lineage 1, Erythroid lineage 2, B cell, and Mast cell) proportion before and after treatment, and healthy donor-derived BM cells are represented at the end of plots. F, The Kaplan-Meier overall survival curves of TCGA AML patients grouped by the cluster-specific gene sets. + represents censored observations, and P value was calculated by multivariate Cox regression. [file 40364_2021_265_MOESM2_ESM.tif]

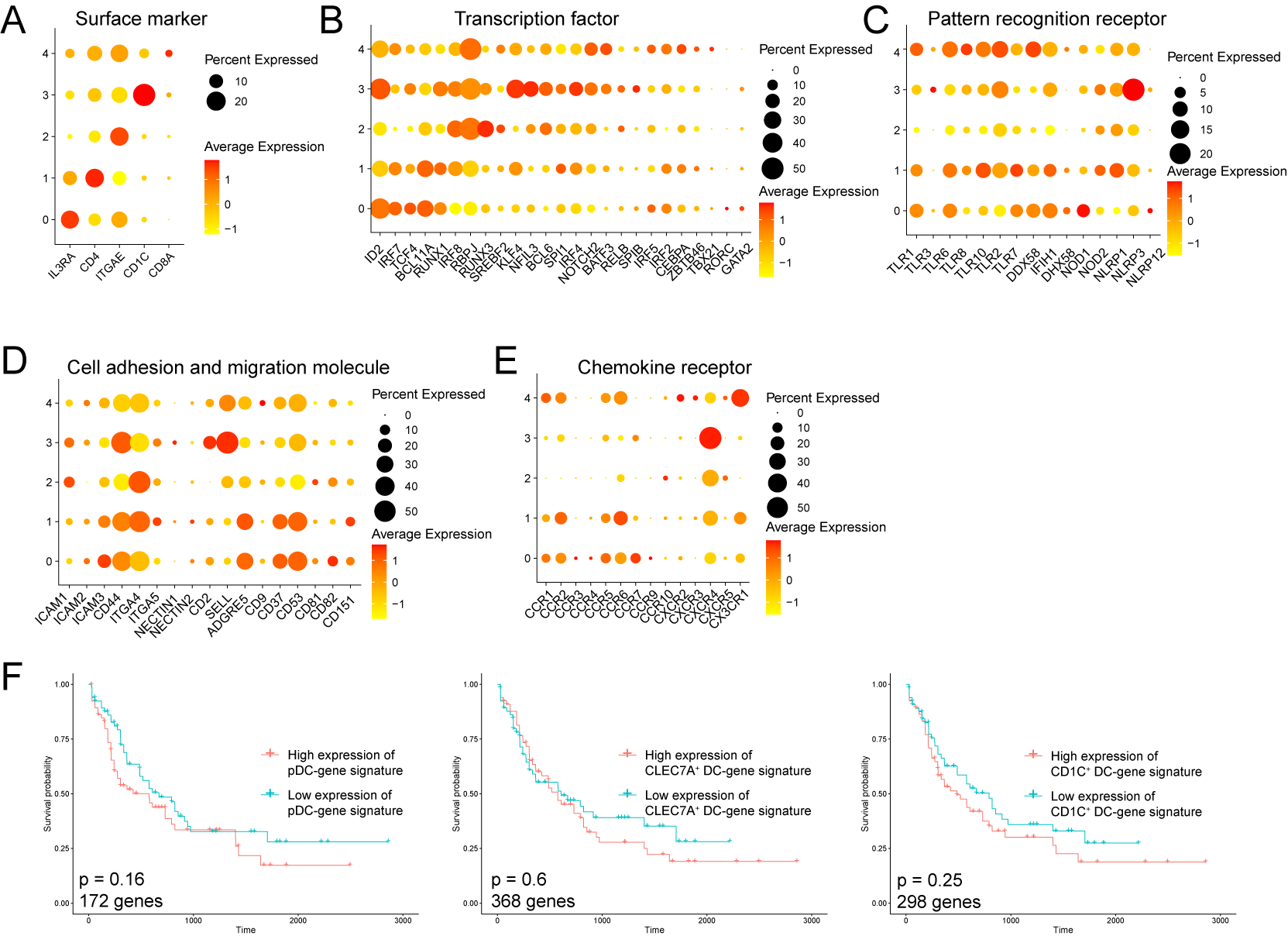

Supplement: Supplementary file 3 — Additional file 3: Supplementary Figure 2. Dot plot of differentially surface markers (A), transcription factors (B), pattern recognition receptors (C), cell adhesion/migration molecules (D), and chemokine receptors (E). F, The Kaplan-Meier overall survival curves of TCGA AML patients grouped by specific DC subset (pDC, CLEC7A+ DC, and CD1C+ DC) gene sets. + represents censored observations, and P value was calculated by multivariate Cox regression. [file 40364_2021_265_MOESM3_ESM.tif]

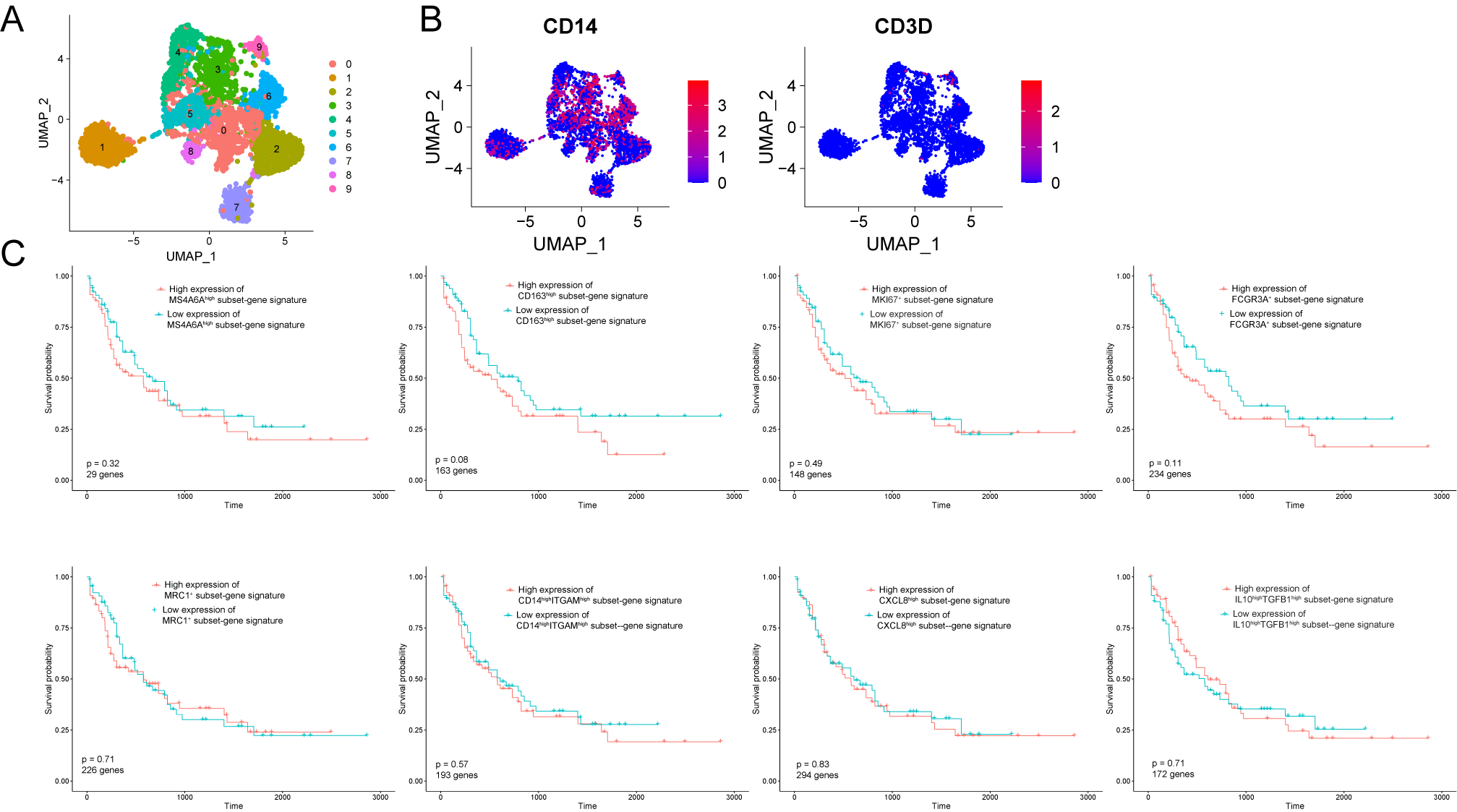

Supplement: Supplementary file 4 — Additional file 4: Supplementary Figure 3. A, UMAP plot of Monocyte/Macrophages from Fig. 1a-represented Mono/Mac cluster. These mature myeloid cells can be divided into 10 subsets before filtering possible cell-cell complexes. B, Expression levels of CD14 and CD3D across Mono/Mac population illustrated in UMAP plots. C, The Kaplan-Meier overall survival curves of TCGA AML patients grouped by specific subset gene sets. + represents censored observations, and P value was calculated by multivariate Cox regression. [file 40364_2021_265_MOESM4_ESM.tif]

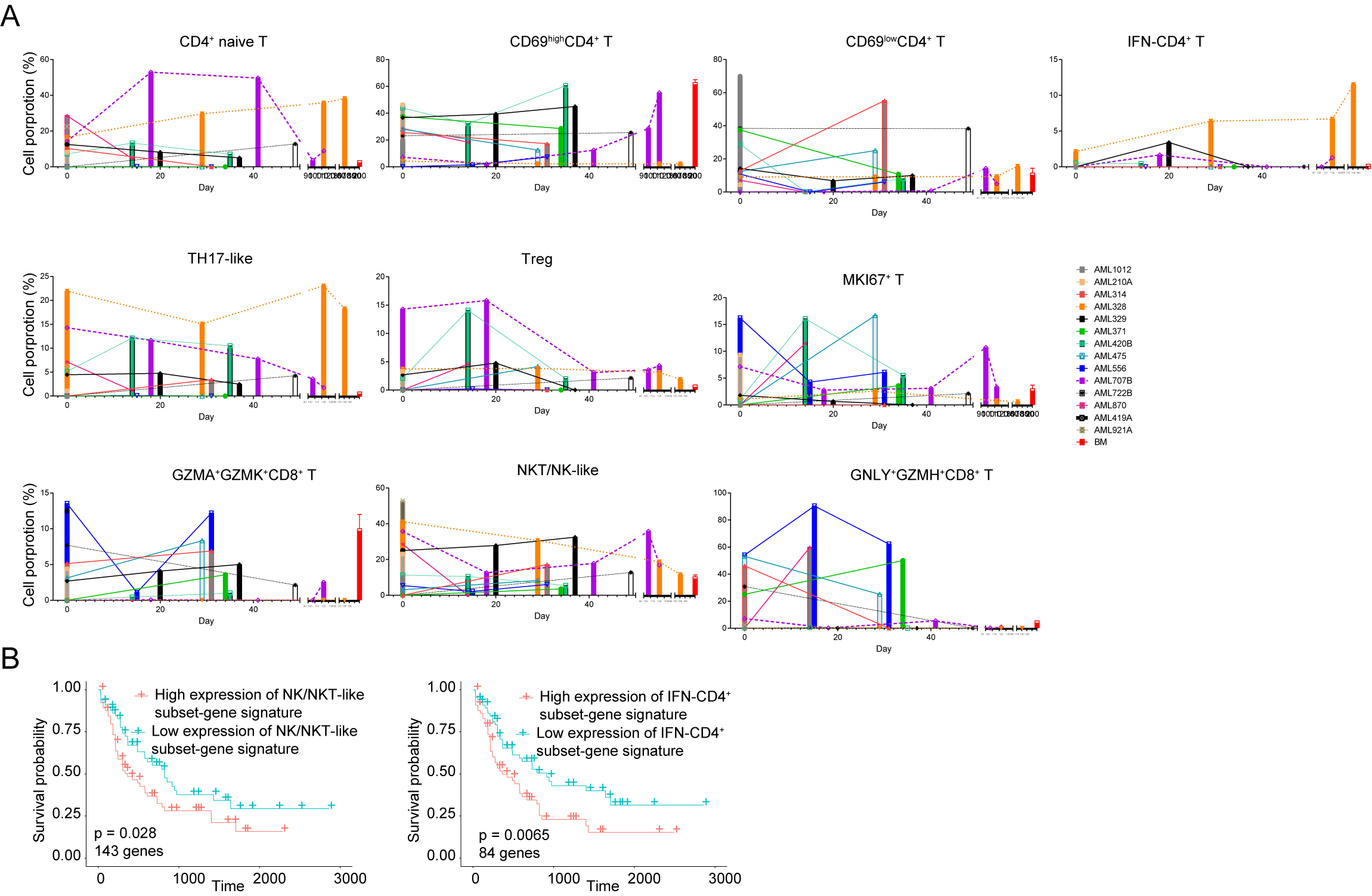

Supplement: Supplementary file 5 — Additional file 5: Supplementary Figure 4. A, dynamic changes of proportion of distinct cell-types in total T/NK cells before and after treatment, and healthy donor-derived BM cells, as control, are represented at the end of plots. B, The Kaplan-Meier overall survival curves of TCGA AML patients grouped by specific NK/NKT-like gene set and IFN-CD4+ gene set. + represents censored observations, and P value was calculated by multivariate Cox regression. [file 40364_2021_265_MOESM5_ESM.tif]

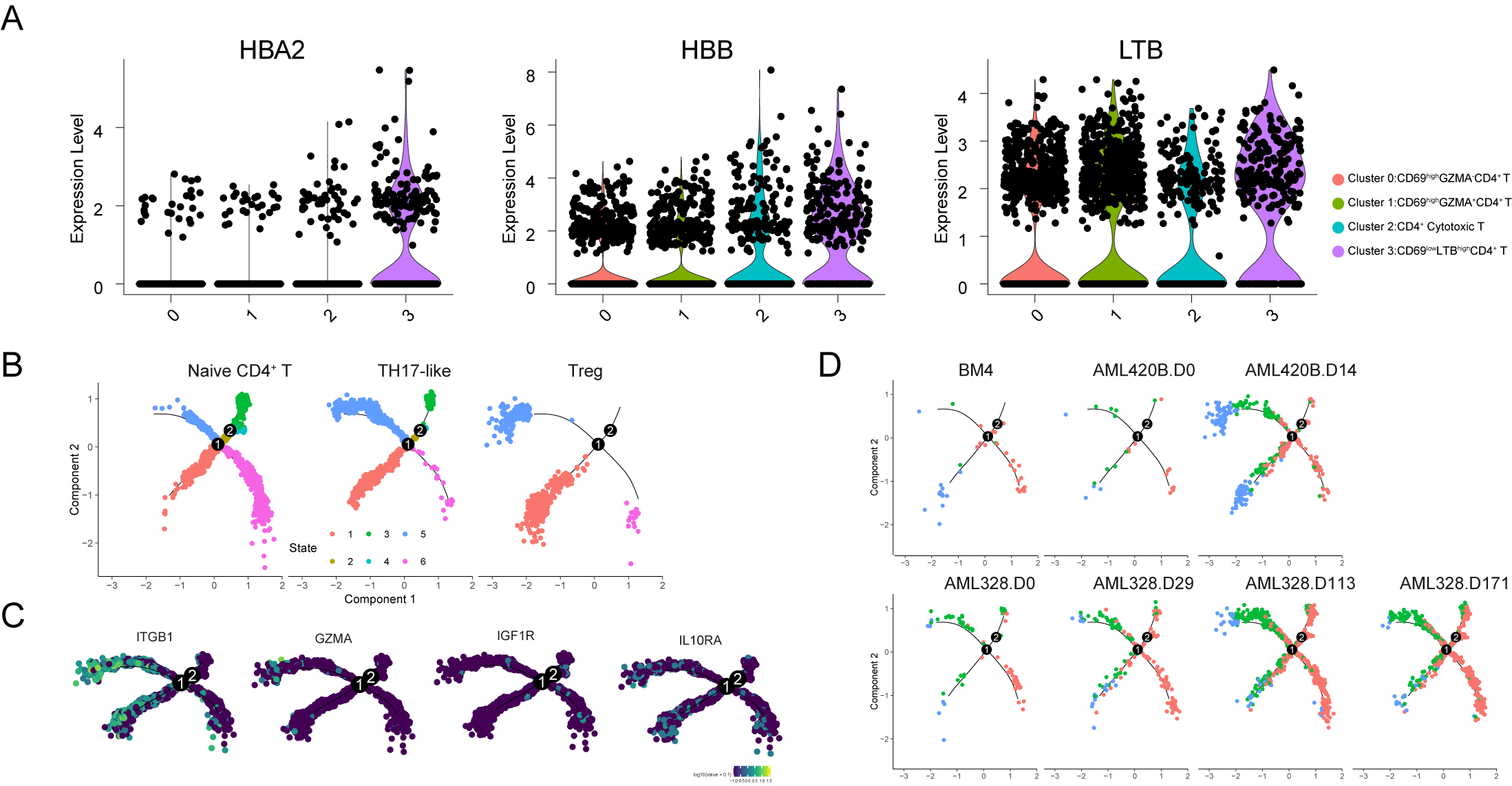

Supplement: Supplementary file 6 — Additional file 6: Supplementary Figure 5. A, Violin plot showing the expression levels of HBA2, HBB, and LTB in 4 clusters (CD69highGZMA-CD4+ T, CD69highGZMA+CD4+ T, CD4+ Cytotoxic T, CD69lowLTBhighCD4+ T) from Fig. 5a-represented cells. B, The state-space of Naïve CD4+ T cluster, TH17-like cluster, and Treg cluster. Each dot corresponded to one single cell, colored according to its state (total 6 states). C, Expression maps showing log-normalized expression of typical markers (ITGB1, GZMA, IGF1R, and IL10RA) in the differentiation of Naïve CD4+ T to TH17-like cells and/or Treg cells. Data are shown as log-normalized expression. Yellow indicates high expression, dark blue indicates low expression. D, Typic state-space of some AML samples (AML420B.D0 and AML420B.D14; AML328.D0, AML328.D29, AML328.D113, and AML328.D171) are represented, and BM4 as healthy control. Each dot corresponded to one single cell, colored according to its state (total 6 states). [file 40364_2021_265_MOESM6_ESM.tif]
